# Mothers’ evaluations of fathers’ contributions to raising children with autism spectrum disorder in the United Arab Emirates

**DOI:** 10.1186/s40359-024-01717-6

**Published:** 2024-05-07

**Authors:** Maxwell Peprah Opoku, Ahmed Mohamed, Mohammed Safi, Shashidhar Belbase, Fadwa Al Mughairbi, Quizhi Xie, Mahmoud Al Shatheli

**Affiliations:** 1 Department of Special and Gifted Education, United Arab Emirates University, Al Ain, Abu Dhabi UAE; 2 Department of Communication Disorders and Deaf Education, Fontbonne University, Minneapolis, Minnesota United States; 3 Mathematics Dept., School of Science and Technology, , Troy, Alabama United States; 4 Department of Clinical Psychology, United Arab Emirates University, Al Ain, Abu Dhabi UAE; 5 Zayed Higher Organisation Al Ain, Al Ain, Abu Dhabi UAE

**Keywords:** Autism spectrum disorder, Involvement, Parenting, Fathers, Mother, United Arab Emirates

## Abstract

**Background:**

Autism spectrum disorder (ASD) is a lifelong neurological condition which results in social skill deficits, communication difficulties, and restrictive and repetitive behaviour. The difficulties associated with parenting children with ASD have been studied extensively, mainly from the perspectives of mothers. The extent of involvement of fathers in the raising of children with ASD has received limited scholarly attention, especially in non-Western contexts such as the United Arab Emirates.

**Objectives:**

This study asked mothers to evaluate the involvement of fathers in the development of children with ASD.

**Methods:**

In all, 240 mothers completed the Fathers’ Involvement in Development and Rehabilitation Scale, designed based on a review of literature on the construct of involvement, namely attitude, participation in training, and support domains. The data were subjected to computation of mean scores, multivariate analysis of variance, hierarchical regression, and moderation analyses.

**Results:**

The results suggested that fathers held positive attitudes and provided substantial support to their children with ASD. However, mothers were ambivalent regarding the participation of fathers in training to support the development of their children. Differences were also observed between participants according to marital status, location, child gender, and ASD severity.

**Conclusion:**

Recommendations for targeted training for fathers and other study implications are discussed.

**Supplementary Information:**

The online version contains supplementary material available at 10.1186/s40359-024-01717-6.

## Introduction

Parental involvement in raising children is acknowledged globally as a moral and human right. In this study, involvement is a multifaceted concept operationalized as the interaction between three related variables: affection, relation or interactions, and actions taken by fathers to support the development [1] of their children with autism spectrum disorder (ASD). This translates into fathers’ acceptance (attitudes), participation in training and supporting the development of their children with ASD [2]. In almost all countries, parental involvement in raising children is safeguarded in national laws and policies [3]. Beyond legal mandates, in every country including the United Arab Emirates (UAE), it is a moral obligation of parents to support the development of children. However, the birth of children with disabilities such as those living with ASD is unexpected and impacts family functioning and cohesion [4–6]. In view of this, scholarly attention has been devoted to the experiences of parents raising children with ASD [5, 6] and their needs [5], identification [7], access to services [8–11] and quality of life [8, 12]. However, in non-Western contexts such as the UAE, studies on the role of fathers in raising children with ASD are very rare or non-existent.

ASD is a neurological disorder characterized by three core features: challenges associated with social skills, communication difficulties and restrictive, and repetitive behaviour [13, 14]. ASD is usually diagnosed at the age of 3 years, and is more prevalent among boys than girls [15]. While the cause of ASD is unknown [13], recent estimates have shown an increase in its prevalence [16]. For instance, in the U.S., the Centre for Disease Control estimated that in 2000, 1 in every 100 children was living with ASD; in 2018, 1 in every 54 children and, in 2023 [17], 1 in every 36 children were living with ASD [15]. In the UAE, according to Virolainen et al. [18], 1 in every 146 children lives with ASD– an estimate projected to increase because of environmental factors. The challenges encountered by children with ASD cannot be overemphasized, and they require substantial support from both fathers and mothers to facilitate their participation in society [3, 19–21]. However, mothers are more likely to be involved in their development [6, 22, 23], which raises questions regarding the extent of involvement of fathers in nurturing and caring for children with ASD.

Studies on the involvement of fathers in the development of children is gaining scholarly attention. For instance, it has been reported that the active involvement of fathers in the raising of typically developing children and those with ASD has positive impacts on the cognitive, social, and emotional development of children [3, 24]. Mainly in Western literature, fathers of children with ASD play myriad roles in the nurturing of their children. This includes but is not limited to advocacy [3, 21], teaching self-care [25, 26], sharing caregiving responsibilities with the spouse [16], financial support [3, 27], communal participation [26] and teaching their children with ASD at home [21, 28]. In non-Western contexts such as the UAE, there is a lack of empirical study on paternal involvement in the raising of children with ASD.

A large body of literature has reported the experiences of fathers raising children with ASD. Both positive [14, 20, 22, 23, 29, 30] and negative [19, 31–38] parenting experiences have been reported. First, fathers have reported that the birth of children with ASD has enhanced their own development and resilience as well as enabled them to be more empathetic [14, 22, 23, 30]. Other studies have reported that the onset of ASD in children contributes to family unity as mothers and fathers unite to raise these children [22, 25]. Conversely, other studies have reported that fathers encounter numerous challenges in raising children with ASD. These challenges include stress, financial problems, difficulty balancing work with parenting, marital problems, and inaccessible services [14, 20, 22, 23, 29, 30]. As a result, fathers have expressed needs such as for training, financial support, and access to essential services [32, 36].

All these studies describe self-reported experiences of fathers pertaining to their roles, responsibilities, and parenting challenges. Since both fathers and mothers are actively involved in the raising of children with ASD, mothers too ought to be given the opportunity to assess the involvement of fathers in the development of their children. Reporting mothers’ ratings of their spouses’ involvement would extend previous studies, which have mainly focused on the self-reported experiences of fathers raising children with ASD.

## Research context

The study reported here was conducted in the UAE. The UAE is made up of seven Emirates: Abu Dhabi, Ajman, Dubai, Fujairah, Ras Al Khaimah, Sharjah and Umm Al Quwain [39]. The UAE is home to an estimated 9.7 million people. The UAE is a cultural and religious society with a sizeable part of the population being Muslims. [40], [39, 41]. [5, 6].

 [4, 42, 43]. children with ASD are perceived as a burden and individuals who require lifelong assistance from other members of the society [4]. Consequently, people are reluctant to associate or contribute towards the development of children with ASD [4, 43]. There are also situations where some families hide their members with ASD from public view and thus, may not seek for help for their children with disabilities [4]. Consequently, the UAE government has taken steps to address this misconception. Most importantly, laws and policies have been formulated and international agreements ratified to create a conducive environment for the development of children with ASD [44, 45]. Nevertheless, parents continue to face challenges in raising children with ASD [4–6]. To better guide the UAE government policies and reforms, there is a need for exploration of the involvement of fathers in the nurturance of children with ASD from the perspectives of mothers, who are, culturally, the primary caregivers of children.

The study reported here used Bogossian et al. [1] and Pleck’s [2] conception of parental involvement as study lens to develop insight into paternal involvement in the raising of children with ASD. It was suggested that parental involvement is a product of three related concepts: attitudes, support, and participation in training. The following research questions were answered in this study:

- What is the extent of involvement (attitude, support, and participation in training) of fathers in raising children with ASD in the UAE?
- What mother/child-related variables impact the involvement (attitude, support, and participating in training) of fathers in raising children with ASD in the UAE?
- Do attitude and participation in training predict fathers’ support of their children with ASD in the UAE?

## Methods

### Study participants

Participants were recruited nationally to develop a broad understanding of fathers’ involvement in raising children with ASD.. The majority of participants were recruited through text message that was sent by the funding institution of this research (Abu Dhabi Early Childhood Authority). This was the largest drive for recruiting mothers and fathers of children with disabilities in Abu Dhabi. Moreover, Zayed Higher Organization for People of Determination and Ministry of Community Development assisted in sharing the survey with parents in Abu Dhabi, Dubai, and Northern Emirates.The inclusion criteria for this study were as follows: (a) mothers raising one or more children with ASD; (b) the child with ASD is either enrolled in school or receiving services in a rehabilitation centre; (c) the child has received a formal diagnosis of ASD; and (d) capacity to consent to participate in the study.

Overall, 240 mothers evaluated their spouses’ involvement in the raising of children with ASD. While 57% were citizens, 43% were residents working in the UAE (see Table 1 for details).

### Instrument

A two-part instrument was used for data collection from mothers. The first part collected background data about the participants (see Table 1 for details).

The second part was the 59-item Fathers’ Involvement in Development and Rehabilitation Scale (FIDRS, see Appendix A), which was developed for this study to assess fathers’ involvement in raising children with ASD. The instrument was developed based on an extensive review of literature [14, 16, 20, 22, 23, 27, 29, 30] on each of the tenets which informed design of the items. The study was guided by three interrelated variables which explains parental involvement (attitudes, support, and training). The literature on each of the tenets of parental involvement were compiled or informed the development of the instrument.

The instrument comprises three domains [support domain (*n* = 37), attitude towards parenting (*n* = 15) and participating in rehabilitation and training (*n* = 7)]. The support domain is made up of three sub-scales (personal support, learning and development, and well-being and development); attitude has two sub-scales (belief towards parenting and beliefs towards support); training is a unidimensional scale. The FIDRS is anchored on a 5-point Likert scale with responses ranging from strongly disagree (1) to strongly agree (5). A composite mean (sum mean divided by the number of items) score of at least 4 was interpreted as favourable involvement of fathers in the development of their children with ASD.

**Table 1:** Summary of demographic characteristics of participants

Category (*N* = 240)	Frequency	Percentage (%)
**Age**		
21–30 years 31–40 years 41 years and above	30 127 83	13% 53% 34%
**Marital status**		
Married Single	216 24	90% 10%
**Nationality**		
Citizens Residents	137 103	57% 43%
**Education level**		
Secondary and below Bachelor or above	117 123	49% 51%
**Employment status**		
Unemployed Employed	155 85	65% 35%
**Monthly family income**		
Less than AED 10,000 AED10,000– AED20,000 AED 21,000 or above	105 57 78	44% 24% 32%
**Number of children with ASD (n = 120)**		
One child with ASD At least two	102 18	85% 15%
**Years of marriage (n = 219)**		
1–5 years 6–10 years 11–15 years or above 16 years or above	105 60 33 21	48% 27% 15% 10%
**Age of children with ASD (n = 233)**		
1–12 years 13–17 years At least 18 years (adults)	176 31 26	76% 13% 11%
**Gender of children with ASD**		
Male Female	188 52	78% 22%
**Severity of ASD**		
Mild Moderate Severe	52 153 35	22% 64% 15%
**Support needs (n = 141)**		
Minimal support Moderate support Substantial support	15 53 73	11% 37% 52%
**School enrolment**		
Yes No	51 90	36% 64%

Before its implementation in this study, the initial draft had 70 items which were subjected to face validation by three experts with experience conducting research on parenting and ASD. Feedback from the experts contributed to trimming the number of items, and the instrument was piloted before its implementation.

The reliability of the scale computed using Cronbach Alpha was as follows: support domain, 0.98 [personal support, 0.90; learning and development, 0.95; well-being and development, 0.96]; attitude towards parenting, 0.95 [belief towards parenting, 0.88; and beliefs towards support, 0.91] and participating in rehabilitation and training, 0.97].

### Procedure

The study and its protocols were approved by the Social Science Ethics Review Committee at UAE University (ERSC_2023_2467). Following institutional approval, approvals were sought from the Emirates Schools Establishment, Zayed Higher Organization for People of Determination, and Ministry of Community Development for permission to collect data from schools and centers across the country. Formal letters were sent to all public schools and rehabilitation centres for permission to conduct this study. Text messages, sent by the funding institution of this study, were the primary way that participants were recruited. Also, some other institutions such as Zayed Higher Organization for People of Determination and Ministry of Community Development were approached to collect data.Disability is sensitive issue [4–6] which makes it difficult to reach mothers. Institutions that responded favourably were sent detailed information statement and online links to be forwarded to mothers for their completion.

The data were collected virtually using *QuestionPro*. The instrument was in both Arabic and English to enable participants to complete it in their preferred language. The data were collected between February 2023 and June 2023. The information statement contained a detailed description of the study, its objectives, and the relevance of the findings to future policy development in the UAE. The participants were assured that neither their identity nor any identifiable information would be used in the reporting of the study. Also, they were assured that the data collected would not be made available to any external body and would be used only for the research purpose. Five participants were randomly selected and given a gift card for participating. All the mothers who participated consented to participating in this study.

### Data analysis

The data collected were transferred to Microsoft Excel for cleaning before being imported to SPSS version 29 for analysis. The normality of the data was observed using boxplots, histograms and Q-Q plots. The data were found to be normally distributed and, as such, supported the use of parametric analysis.

To answer research question 1, mean scores were calculated for each of the tenets/sub-scales. For research question 2, multivariate analysis of variance (MANOVA) was performed to understand the differences between participants in the combined dependent variables (attitude, support, and participating in training) [46]. The following assumptions were observed to make sure that they were not violated: linearity, outliers and homogeneity of variance. A Bonferroni-adjusted alpha level of 0.01 (i.e., 0.05 divided by 3, which is the number of dependent variables) [46] was the baseline for determining whether there were differences between the participants. The strength of the difference was assessed using the effect size (partial eta squared), which was interpreted as follows: small (0.01–0.05), moderate (0.06–0.1) and large (at least 0.1) [46].

Before answering research question 3, initial Pearson moment correlation coefficients were computed to check the relationship between attitude, support, and participation in training. The strength of the relationship was interpreted as follows: small (0.10–0.30), moderate (0.31–0.50) and large (at least 0.51) [46]. Following this, hierarchical regression was computed to explore the contributions of attitude and participation in training to support. While attitude and participation in training were entered in step 1, demographic variables were entered in step 2. The following assumptions were observed to make sure they were not violated: normality, linearity, multicollinearity, and homoscedasticity [46].

Afterwards, the demographic variables (used as moderators) which made significant contributions in the variance in support were assessed further using Andrew Hayes’ moderation model method 1 [47]. This is to help understand the interaction effect of demographic variables on the relationship between the independent and dependent variables.

## Results

The mean scores for the scale were as follows: support domain, *M* = 4.11, *SD* = 0.77; [personal support, *M* = 4.06, *SD* = 0.74; learning and development, *M* = 4.32, *SD* = 0.86; and well-being and development, *M* = 3.95, *SD* = 0.84]; attitude towards parenting, *M* = 3.86, *SD* = 0.81 [belief towards parenting, *M* = 3.94, *SD* = 0.85; and beliefs towards support, M = 3.81, *SD* = 0.83], and participating in rehabilitation and training, *M* = 3.74, *SD* = 0.60].

### Differences between participants

MANOVA was conducted to explore the differences between participants on the dependent variables (see Table 2). First, a difference was observed between participants regarding marital status for the combined dependent variables, *F*(3, 236) = 7.87, *Wilks’ lambda* = 0.91, *p* =.001, with a large effect size, *partial eta squared* = 0.09. Individually, differences were found between participants in the support domain [*F*(1, 238) = 18.88, *p* =.001, with a large effect size, partial eta squared = 0.07] and in attitude towards parenting children with ASD [*F*(1, 238) = 20.36, *p* =.001, with a large effect size, *partial eta squared* = 0.08]. Observation of the mean scores suggested that participants who indicated they were married also indicated more support and more favourable attitude of fathers towards parenting children with ASD [support domain, married, *M* = 4.18, *SD* = 0.63; single, *M* = 3.49, *SD* = 1.44; attitude, married, *M* = 3.94, *SD* = 0.67; single, *M* = 3.18, *SD* = 1.42].

**Table 2:** Difference between participants

	Wilks’ Lambda	MAN. F	ANOVA F		
			Support	Attitude	Training
**Age** Effect size	0.97	1.14 0.01	1.90 0.02	0.97 0.008	1.15 0.01
**Marital status** Effect size	0.91	7.87** 0.09	18.88** 0.07	20.36** 0.08	0.40 0.002
**Nationality** Effect size	0.99	0.26 0.003	0.73 0.003	0.54 0.002	0.13 0.001
**Education level** Effect size	0.99	0.37 0.005	0.83 0.003	0.27 0.001	0.02 0.001
**Employment status** Effect size	0.99	0.34 0.004	0.16 0.001	0.14 0.001	0.96 0.004
**Monthly family income** Effect size	0.97	1.41 0.02	1.43 0.01	0.60 0.005	0.89 0.007
**Number of children with ASD** Effect size	0.99	0.36 0.009	0.69 0.006	0.84 0.007	0.41 0.003
**Years of marriage** Effect size	0.96	1.00 0.01	1.01 0.01	0.58 0.008	1.19 0.02
**Age of children with ASD** Effect size	0.95	2.04 0.03	3.26 0.03	1.66 0.01	0.30 0.003
**Gender of children with ASD** Effect size	0.96	3.60** 0.04	0.59 0.002	3.45 0.01	5.77* 0.02
**Severity of ASD** Effect size	0.96	1.58 0.02	2.15 0.02	0.87 0.007	1.39 0.01
**Support needs** Effect size	0.95	1.24 0.03	1.68 0.02	1.63 0.02	1.22 0.02
**School enrolment** Effect size	0.99	0.33 0.007	0.09 0.001	0.04 0.001	0.10 0.001

**p ≤.01; *p ≤.05

Second, a difference was observed between participants for the combined dependent variables, *F*(3, 236) = 3.60, *Wilks’ lambda* = 0.96, *p* =.01, with a moderate effect size, *partial eta squared* = 0.04. Individually, a difference was found between participants regarding rehabilitation and training, *F*(1, 238) = 5.76, *p* =.02, with a small effect size, *partial eta squared* = 0.02. The mean scores showed fathers with male children with ASD (*M* = 3.79, *SD* = 0.58) were more likely to engage in training than those with female children with ASD (*M* = 3.56, *SD* = 0.60).

### Predictors of fatherly involvement

The relationships between the three domains were computed for Pearson moment correlation coefficients: support provided to children with ASD and attitude (*r* =.85, *p* =.001); support provided to children with ASD and rehabilitation and training (*r* =.17, *p* =.01); attitude towards parenting and rehabilitation and training (*r* =.16, *p* =.02).

**Table 3:** Attitude and training regressed on fathers’ support to children with ASD

	Uns. Beta	Stand. Error	Stand. Beta	t	p
**Step 1**					
Attitude towards training	2.01	0.11	0.86	18.26	0.001**
Rehabilitation and training	0.226	0.38	0.03	0.59	0.56
**Step 2**					
Attitude towards training	1.97	0.12	0.85	16.39	0.001**
Rehabilitation and training	0.14	0.41	0.07	0.34	0.73
Age	− 0.85	3.36	− 0.04	− 0.25	0.80
Marital status	-5.30	5.91	− 0.05	− 0.90	0.37
Nationality	-8.84	4.54	− 0.11	-1.95	0.06
Education level	5.46	4.01	0.07	1.36	0.18
Employment status	-6.51	4.02	− 0.08	-1.62	0.11
Monthly family income	-2.18	2.48	− 0.05	− 0.88	0.38
Number of children with ASD	-2.74	5.46	− 0.03	− 0.50	0.62
Years of marriage	− 0.43	2.06	− 0.01	− 0.21	0.83
Age of children with ASD	1.50	2.61	0.03	0.58	0.57
Gender of children with ASD	2.09	5.00	0.02	0.42	0.68
Severity of ASD	-7.96	3.81	− 0.13	-2.09	0.04*
Support needs	1.93	3.35	0.04	0.58	0.57
School enrolment	-6.31	3.82	− 0.08	-1.65	0.10

^**^p ≤.01; ^*^p ≤.05

Hierarchical regression was performed to explore the contributions of attitude and training to the variance in support provided to children with ASD (see Table 3). In step 1, attitude and training made significant contributions in the variance in fatherly support for children with ASD, *F*(2, 116) = 172.41, *p* =.001. They together contributed 75% to the variance in support for children with ASD. However, individually, only attitude towards parenting children with ASD (*β* = 0.86, *p* =.001) made a significant contribution to the variance in support for children with ASD.

In step 2, demographic variables were added to the model. The demographic variables made a nonsignificant contribution of 4% to the variance in support provided to children with ASD, *F*(13, 102) = 1.49, *p* =.13.

The combination of demographic variables and independent variables made a significant contribution of 79% in the variance in support for children with ASD, *F*(15, 118) = 24.14, *p* =.001. Individually, two predictors made significant contributions in support for children with ASD: attitude towards parenting children with ASD (*β* = 0.85, *p* =.001) and severity of ASD (*β* = −0.13, *p* =.04). Overall, attitude towards parenting children with ASD made the most significant contribution in the variance in support provided to children with ASD.

### Moderators of relationship between attitude and support

**Fig. 1:**
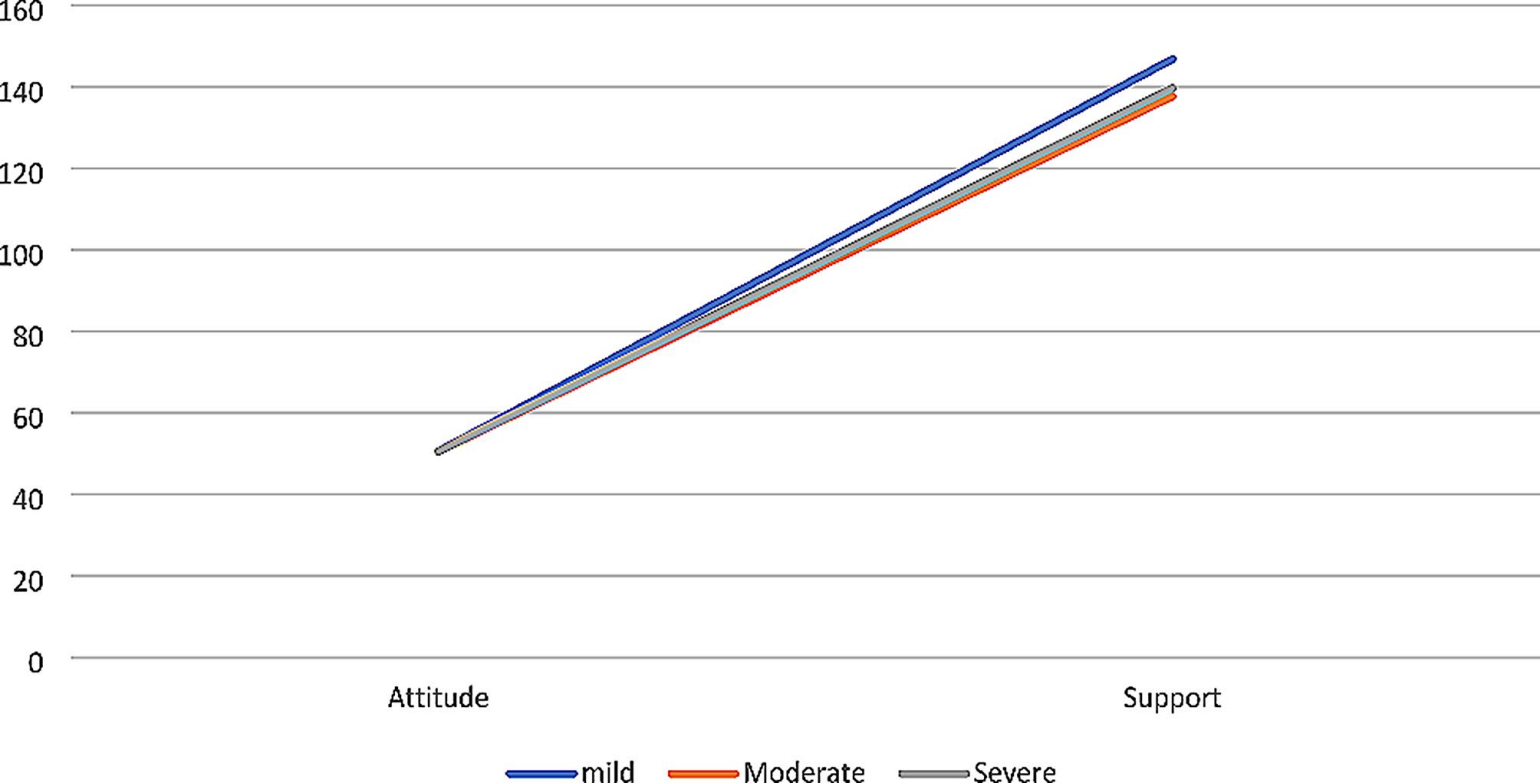
Severity of ASD as moderator of attitude and support

Since severity of ASD impacted the support domain, its interaction effects on the relationship between the attitude and support domains were examined. Severity of ASD significantly moderated the relationship between attitude and support, *β* = 0.64, 95% CI (0.41, 0.87), *t* = 5.44, *p* =.001. In the event participants had minimal support [*β* = 1.34, 95% CI (1.07, 1.62), *t* = 9.58, *p* =.001], moderate support [*β* = 1.98, 95% CI (1.83, 2.13), *t* = 25.79, *p* =.001] or substantial support [*β* = 1.91, 95% (1.67, 2.88), *t* = 25.08, *p* =.001], a significant relationship was seen between attitude and support. As Fig. 1 indicates, children who living with mild ASD received higher support domain than those with moderate or severe ASD.

## Discussion

In this study, mothers evaluated the involvement of their spouses in the raising of children with ASD. The study was conducted against the backdrop of mothers being the primary carers for their children with ASD in non-Western contexts such as the UAE. The findings showed that the fathers appear to embrace their children as well as support their development. However, the fathers appear to fall short when it comes to participating in training programmes to enhance the development of their children. The findings are consistent with those of previous studies which found positive attitude and fathers’ support for their children with ASD [14, 20, 22, 23, 29, 30]. The findings are also consistent with previous studies which reported fathers’ concerns regarding access to training for the purpose of learning new skills to support their children [14, 31, 32, 35]. The low ratings of participants on fathers’ participation in training could be linked to their work schedules. However, the involvement of fathers is vital to the development of their children [3, 19, 35]. In view of this, policymakers could consider developing flexible online training modules specifically for fathers of children with ASD. High-achieving fathers could be celebrated to encourage others to participate in such training.

The findings indicated a significant relationship between attitude and supporting the development of children with ASD. The computation of correlation and hierarchical regression showed a strong relationship between attitude and support domains. This finding is expected in a context such as the UAE, where there is a cultural interpretation given to the onset of ASD as well as to the support and development of children with ASD [4, 42, 43]. The findings reported here show that to promote the involvement of fathers in the development of children with ASD, deliberate efforts should be made to change their attitude and embrace such children as equal members of society. In the literature, fathers seem to struggle in the event their children are diagnosed with ASD [14, 22, 36, 37]. While their situation can contribute to the resilience of fathers [20, 22, 30, 32], nonetheless it sometimes breaks the family [22, 37]. In the UAE context, training and counselling programmes ought to be developed for fathers before and after their children’s diagnoses. The training could focus on the aetiology of ASD and opportunities available in society for children with ASD.

Demographic variables provided additional insight into fathers’ involvement in the nurturance of their children with ASD. For example, marital status of parents exhibited a difference between participants regarding the support domain and attitudes. Mothers who indicated that they were married rated their spouses as more supportive with a favourable disposition towards children with ASD than those who indicated they were single (e.g. divorced, separated). This is an interesting finding because the onset of ASD contributes to divorce, low family functioning, and tension within families [5, 22, 29, 37, 48, 49]. Although this study did not gather information about the cause of the separation or divorce between parents, the diagnosis of children with ASD could play a pivotal role. Due to tension between spouses, children might not get the support they need from their fathers. In the literature, it has been reported that mothers were at high risk of stress and poor quality of life in the event they have to shoulder all caregiving responsibilities [38]. Policymakers may prioritize parental counselling and contributions towards the raising of children with ASD. There should also be social support programmes designed for single mothers raising children with ASD in the UAE.

The severity of ASD emerged as a moderator of the relationship between attitude and support. In this respect, while mothers did not differ on attitudes towards children with ASD, their reports differed regarding the support fathers provided to children with ASD. It is apparent that the greater the severity of the disability, the less involved fathers were in raising their children with ASD. This finding may not be surprising because other studies have reported the severity of ASD as a major source of stress or concern to fathers of children with ASD [34, 38]. In most societies, children with ASD are considered a burden to not only families but to society as whole [4, 12]. Society also focuses more on their weakness and not their strength or potential contribution [43]. There is the possibility that some fathers shared similar perspectives and did not offer much assistance in the event their children were diagnosed as having severe ASD. Undoubtedly, there is strength in disability, and fathers could be made aware of this strength and their role in nurturing the potential of children with ASD. Going forward, policymakers could consider embarking on intense public education and engagement with fathers to enable them understand their children’s uniqueness and potential.

### Study limitations

The study reported here is not without limitations. First, the participants were skewed towards those who had enrolled their children with ASD in special schools/centers or who were receiving services at rehabilitation centres. The mothers of children with ASD outside these settings were excluded and, thus, it is impossible to generalize the findings. However, there are common systems and a shared culture between those who took part in this study and those who were excluded. The findings reported here could mirror the experiences of mothers who were considered for participation. Second, the study was guided by self-reported experiences of mothers and thus is susceptible to response bias. More so, it was beyond the scope of this study to verify the claims reported by participants. Mothers were provided an information statement, and they rated fathers’ involvement in their preferred language of choice or fluency. There is potential that they provided accurate accounts of their spouses’ involvement in the raising of children with ASD. Notwithstanding, a future study could draw on fathers to understand their involvement in the rehabilitation of children with ASD. This could provide a clearer picture of the extent of fathers’ involvement in raising children with ASD.

### Conclusion and implications for practice

The study reported here presents mothers’ evaluations of their spouses’ involvement in the raising of children with ASD in the UAE. To the best of our knowledge, this is the first time the role of fathers in raising children with ASD has been studied in a non-Western context such as the UAE. The results showed a high level of support and favourable attitudes among fathers towards raising children with ASD. However, the mothers who took part in this study were ambivalent regarding the participation of fathers in training to support the development of their children with ASD. Additionally, there was a high correlation between fathers’ attitude and supporting the development of their children with ASD. Finally, differences were reported for marital status, the gender of children, location, and severity of disability.

The findings are promising and provide an important direction which could be considered by policymakers in the UAE. First, the findings showed apparent low participation of fathers in training programmes. This underscores the need for policymakers to consider developing targeted training programmes on aetiology, needs, interventions, and services available in the community to children with ASD. Also, targeted and ongoing counselling programmes could be developed for fathers to enhance their understanding and acceptance of children with ASD. Moreover, social support programmes could be institutionalized for single mothers raising children with ASD. This could lessen the burden on mothers and enhance their mental well-being. Similarly, fathers who are divorced or separated could be engaged on the importance of their involvement in the development of children with ASD. The study reported here has extended discussion on fathers’ involvement in raising children with ASD in the UAE, and the suggestions above could be considered by policymakers designing future programmes or policy development.

### Electronic supplementary material

Below is the link to the electronic supplementary material.

Supplementary Material 1

## Data Availability

The datasets used and/or analysed during the current study available from the corresponding author on reasonable request.

## Abbreviations

ASD: Autism Spectrum Disorder
FIDRS: Fathers’ Involvement in Disability and Rehabilitation Scale
UAE: United Arab Emirates
