# Supplementary material for: Mothers’ evaluations of fathers’ contributions to raising children with autism spectrum disorder in the United Arab Emirates
Source: BMC Psychol. 2024 May 7;12:253. doi: 10.1186/s40359-024-01717-6 (PMC11077709; doi:10.1186/s40359-024-01717-6)
Supplement: Supplementary file 1 — Supplementary Material 1 [file 40359_2024_1717_MOESM1_ESM.docx]

Appendix A

Fathers’ Involvement in Development and Rehabilitation Scale

Which best describes you?

1. 1. Father of Child/ren of Determination
2. 2. Mother of Child/ren of Determination
3. 3. Practitioner working with Children of Determination
4. 4. None of the above

Do you have at least one child of determination between the ages of 0 and 8?

1. 1. Yes
2. 2. No

Do you have at least one child of determination between the ages of 0 and 8?

1. 1. Yes
2. 2. No

Dear Father,A research team from United Arab Emirates University is conducting a research study about fathers’ role in raising children of determination within a funded research project from Abu Dhabi Early Childhood Authority (ECA) with the intention to provide greater support to fathers of children of determination by better understanding your views and needs. The purpose of this survey is to explore your perceptions about raising children of determination so that they reach their full potential. The first part of the survey consists of some demographic information. The second part consists of some information about your child. The third part includes questions about your interactions with your child of determination. Answering the survey will take about 15 minutes. Thanks for your time.We understand that your time is valuable, and we appreciate your willingness to participate in this survey. As a token of our gratitude, we will ask for your email address at the end of the survey, if you would like to be entered into a draw to receive a gift voucher of AED 500 as a thanks for participating. Please note that you are not required to submit your email address and this survey remains completely anonymous. Consent·      I acknowledge that I have read and understood the information about the study. ·      I understand that my participation is voluntary, and I can withdraw at any time.·      I understand that this information is confidential and that any publications coming out of this research will protect the confidentiality of all participants.

Consent Form

1. 1. I would like to take part in this study and give my informed consent to participate
2. 2. I would NOT like to take part in this study

Dear Mother,A research team from United Arab Emirates University is conducting a research study about fathers’ role in raising children of determination within a funded research project from Abu Dhabi Early Childhood Authority (ECA). The purpose of this survey is to explore fathers’ perceptions about raising children of determination in the family to promote their roles to cater and serve them to reach their full potential. The   first part of the survey consists of some demographic information. The second part consists of some information about your child. The third part includes your understanding of your husband’s practices with your child. Kindly answer the survey items as to your observations of your husband’s interactions with your child. Answering the survey will take about 15 minutes. Thanks for your time.We understand that your time is valuable, and we appreciate your willingness to participate in this survey. As a token of our gratitude, we will ask for your email address at the end of the survey, if you would like to be entered into a draw to receive a gift voucher of AED 500 as a thanks for participating. Please note that you are not required to submit your email address and this survey remains completely anonymous. Consent·      I acknowledge that I have read and understood the information about the study. ·      I understand that my participation is voluntary, and I can withdraw at any time.·      I understand that this information is confidential and that any publications coming out of this research will protect the confidentiality of all participants.

Consent Form

1. 1. I would like to take part in this study and give my informed consent to participate
2. 2. I would NOT like to take part in this study

Dear COD Practitioner,A research team from United Arab Emirates University is conducting a research study about fathers’ role in raising children of determination within a funded research project from Abu Dhabi Early Childhood Authority (ECA). The purpose of this survey is to explore practitioners’ perceptions about fathers’ roles in raising children of determination perceptions about raising children of determination to support them in reaching their full potential. The first part of the survey consists of some demographic information. The second part asks about your understanding of fathers’ practices with their child of determination. Answering the survey will take about 10 minutes. Thanks for your time.We understand that your time is valuable, and we appreciate your willingness to participate in this survey. As a token of our gratitude, we will ask for your email address at the end of the survey, if you would like to be entered into a draw to receive a gift voucher of AED 500 as a thanks for participating. Please note that you are not required to submit your email address and this survey remains completely anonymous. Consent·      I acknowledge that I have read and understood the information about the study. ·      I understand that my participation is voluntary, and I can withdraw at any time.·      I understand that this information is confidential and that any publications coming out of this research will protect the confidentiality of all participants.

Consent Form

1. 1. I would like to take part in this study and give my informed consent to participate
2. 2. I would NOT like to take part in this study

How old are you?

1. 1. 18
2. 2. 19
3. 3. 20
4. 4. 21
5. 5. 22
6. 6. 23
7. 7. 24
8. 8. 25
9. 9. 26
10. 10. 27
11. 11. 28
12. 12. 29
13. 13. 30
14. 14. 31
15. 15. 32
16. 16. 33
17. 17. 34
18. 18. 35
19. 19. 36
20. 20. 37
21. 21. 38
22. 22. 39
23. 23. 40
24. 24. 41
25. 25. 42
26. 26. 43
27. 27. 44
28. 28. 45
29. 29. 46
30. 30. 47
31. 31. 48
32. 32. 49
33. 33. 50
34. 34. 51
35. 35. 52
36. 36. 53
37. 37. 54
38. 38. 55
39. 39. 56
40. 40. 57
41. 41. 58
42. 42. 59
43. 43. 60
44. 44. 61
45. 45. 62
46. 46. 63
47. 47. 64
48. 48. 65
49. 49. 66
50. 50. 67
51. 51. 68
52. 52. 69
53. 53. 70
54. 54. 71
55. 55. 72
56. 56. 73
57. 57. 74
58. 58. 75
59. 59. 76
60. 60. 77
61. 61. 78
62. 62. 79
63. 63. 80
64. 64. 81
65. 65. 82
66. 66. 83
67. 67. 84
68. 68. 85
69. 69. 86
70. 70. 87
71. 71. 88
72. 72. 89
73. 73. 90
74. 74. 91
75. 75. 92
76. 76. 93
77. 77. 94
78. 78. 95
79. 79. 96
80. 80. 97
81. 81. 98
82. 82. 99

What is your marital status?

1. 1. Married
2. 2. Single
3. 3. Divorced
4. 4. Separated
5. 5. Widowed

What is your primary nationality?

1. 1. Afghanistan
2. 2. Albania
3. 3. Algeria
4. 4. Andorra
5. 5. Angola
6. 6. Antigua and Barbuda
7. 7. Argentina
8. 8. Armenia
9. 9. Australia
10. 10. Austria
11. 11. Azerbaijan
12. 12. Bahamas
13. 13. Bahrain
14. 14. Bangladesh
15. 15. Barbados
16. 16. Belarus
17. 17. Belgium
18. 18. Belize
19. 19. Benin
20. 20. Bhutan
21. 21. Bolivia
22. 22. Bosnia and Herzegovina
23. 23. Botswana
24. 24. Brazil
25. 25. Brunei Darussalam
26. 26. Bulgaria
27. 27. Burkina Faso
28. 28. Burundi
29. 29. Cambodia
30. 30. Cameroon
31. 31. Canada
32. 32. Cape Verde
33. 33. Central African Republic
34. 34. Chad
35. 35. Chile
36. 36. China
37. 37. Colombia
38. 38. Comoros
39. 39. Congo, Republic of the...
40. 40. Costa Rica
41. 41. Côte d'Ivoire
42. 42. Croatia
43. 43. Cuba
44. 44. Cyprus
45. 45. Czech Republic
46. 46. Democratic Republic of the Congo
47. 47. Denmark
48. 48. Djibouti
49. 49. Dominica
50. 50. Dominican Republic
51. 51. Ecuador
52. 52. Egypt
53. 53. El Salvador
54. 54. Equatorial Guinea
55. 55. Eritrea
56. 56. Estonia
57. 57. Ethiopia
58. 58. Fiji
59. 59. Finland
60. 60. France
61. 61. Gabon
62. 62. Gambia
63. 63. Georgia
64. 64. Germany
65. 65. Ghana
66. 66. Greece
67. 67. Grenada
68. 68. Guatemala
69. 69. Guinea
70. 70. Guinea-Bissau
71. 71. Guyana
72. 72. Haiti
73. 73. Honduras
74. 74. Hong Kong (S.A.R.)
75. 75. Hungary
76. 76. Iceland
77. 77. India
78. 78. Indonesia
79. 79. Iran
80. 80. Iraq
81. 81. Ireland
82. 82. Israel
83. 83. Italy
84. 84. Jamaica
85. 85. Japan
86. 86. Jordan
87. 87. Kazakhstan
88. 88. Kenya
89. 89. Kiribati
90. 90. Kuwait
91. 91. Kyrgyzstan
92. 92. Lao People's Democratic Republic
93. 93. Latvia
94. 94. Lebanon
95. 95. Lesotho
96. 96. Liberia
97. 97. Libyan Arab Jamahiriya
98. 98. Liechtenstein
99. 99. Lithuania
100. 100. Luxembourg
101. 101. Madagascar
102. 102. Malawi
103. 103. Malaysia
104. 104. Maldives
105. 105. Mali
106. 106. Malta
107. 107. Marshall Islands
108. 108. Mauritania
109. 109. Mauritius
110. 110. Mexico
111. 111. Micronesia, Federated States of...
112. 112. Monaco
113. 113. Mongolia
114. 114. Montenegro
115. 115. Morocco
116. 116. Mozambique
117. 117. Myanmar
118. 118. Namibia
119. 119. Nauru
120. 120. Nepal
121. 121. Netherlands
122. 122. New Zealand
123. 123. Nicaragua
124. 124. Niger
125. 125. Nigeria
126. 126. North Korea
127. 127. Norway
128. 128. Oman
129. 129. Pakistan
130. 130. Palestine
131. 131. Palau
132. 132. Panama
133. 133. Papua New Guinea
134. 134. Paraguay
135. 135. Peru
136. 136. Philippines
137. 137. Poland
138. 138. Portugal
139. 139. Qatar
140. 140. Republic of Moldova
141. 141. Romania
142. 142. Russian Federation
143. 143. Rwanda
144. 144. Saint Kitts and Nevis
145. 145. Saint Lucia
146. 146. Saint Vincent and the Grenadines
147. 147. Samoa
148. 148. San Marino
149. 149. Sao Tome and Principe
150. 150. Saudi Arabia
151. 151. Senegal
152. 152. Serbia
153. 153. Seychelles
154. 154. Sierra Leone
155. 155. Singapore
156. 156. Slovakia
157. 157. Slovenia
158. 158. Solomon Islands
159. 159. Somalia
160. 160. South Africa
161. 161. South Korea
162. 162. Spain
163. 163. Sri Lanka
164. 164. Sudan
165. 165. Suriname
166. 166. Swaziland
167. 167. Sweden
168. 168. Switzerland
169. 169. Syrian Arab Republic
170. 170. Tajikistan
171. 171. Thailand
172. 172. The former Yugoslav Republic of Macedonia
173. 173. Timor-Leste
174. 174. Togo
175. 175. Tonga
176. 176. Trinidad and Tobago
177. 177. Tunisia
178. 178. Turkey
179. 179. Turkmenistan
180. 180. Tuvalu
181. 181. Uganda
182. 182. Ukraine
183. 183. United Arab Emirates
184. 184. United Kingdom of Great Britain and Northern Ireland
185. 185. United Republic of Tanzania
186. 186. United States of America
187. 187. Uruguay
188. 188. Uzbekistan
189. 189. Vanuatu
190. 190. Venezuela, Bolivarian Republic of...
191. 191. Viet Nam
192. 192. Yemen
193. 193. Zambia
194. 194. Zimbabwe

What is your Emirate of residence?

1. 1. Abu Dhabi
2. 2. Ajman
3. 3. Dubai
4. 4. Fujairah
5. 5. Ras Al Khaimah
6. 6. Sharjah
7. 7. Umm Al Quwain

In which region of Abu Dhabi do you live?

1. 1. Abu Dhabi
2. 2. Al Ain
3. 3. Al Dhafra

What is the highest level of education you have attained?

1. 1. Ability to read and write
2. 2. Completed primary school
3. 3. Completed High School/Secondary School
4. 4. Completed Bachelor&#39;s Degree
5. 5. Completed Masters Degree
6. 6. Completed Doctoral Degree
7. 7. Other __________

Which of the following best describes your employment status of the last 12-months

1. 1. Full time employment
2. 2. Part time employment
3. 3. Unemployed
4. 4. Self-employed
5. 5. Retired
6. 6. Student
7. 7. Unpaid/Volunteer
8. 8. Homemaker

What is the total household monthly income (including all family members in your household)?

1. 1. Less than AED 10,000
2. 2. AED 10,000 – AED 20,000
3. 3. AED 20,001 – AED 30,000
4. 4. AED 30,001 – AED 40,000
5. 5. Above AED 40,000

For how many years were you married when your first child of determination was born?

1. 1. Less than one year
2. 2. 1
3. 3. 2
4. 4. 3
5. 5. 4
6. 6. 5
7. 7. 6
8. 8. 7
9. 9. 8
10. 10. 9
11. 11. 10
12. 12. 11
13. 13. 12
14. 14. 13
15. 15. 14
16. 16. 15
17. 17. 16
18. 18. 17
19. 19. 18
20. 20. 19
21. 21. 20
22. 22. 21
23. 23. 22
24. 24. 23
25. 25. 24
26. 26. 25
27. 27. 26
28. 28. 27
29. 29. 28
30. 30. 29
31. 31. 30
32. 32. 31
33. 33. 32
34. 34. 33
35. 35. 34
36. 36. 35
37. 37. 36
38. 38. 37
39. 39. 38
40. 40. 39
41. 41. 40
42. 42. More than 40 years

Please answer these questions about your child of determination. If you have more than one child of determination, please answer the following questions relating to ONE child who is between 0 and 8 years old.

What is your child’s age?

1. 1. Under 1 year
2. 2. 1
3. 3. 2
4. 4. 3
5. 5. 4
6. 6. 5
7. 7. 6
8. 8. 7
9. 9. 8
10. 10. Above 8 years old

What is your child&#39;s gender?

1. 1. Female
2. 2. Male

At what age was your child diagnosed as a child of determination?

1. 1. 0-3 months
2. 2. 3-6 months
3. 3. 6-9 months
4. 4. 9-12 months
5. 5. 1 years old
6. 6. 2 years old
7. 7. 3 years old
8. 8. 4 years old
9. 9. 5 years old
10. 10. 6 years old
11. 11. 7 years old
12. 12. 8 years old
13. 13. They have not received a formal diagnosis

Which of the following relates to your child&#39;s diagnosis?

1. 1. Intellectual disability
2. 2. Autism Spectrum Disorder
3. 3. Communication Disorders
4. 4. Physical/Motor Disability
5. 5. Hearing impairment/deafness
6. 6. Visual impairment/blindness
7. 7. Cerebral palsy
8. 8. Other

How would you rate the level of your child’s disability?

1. 1. Mild
2. 2. Moderate
3. 3. Severe

How would you rate the level of support that your child needs?

1. 1. They require minimal support
2. 2. They require moderate support
3. 3. They require substantial support

Does your child of determination attend school?

1. 1. Yes
2. 2. No

Does your child of determination attend a Rehabilitation Center?

1. 1. Yes
2. 2. No

Does your child participate in any intervention programs (such as physiotherapy, speech therapy, etc.)?

1. 1. Yes
2. 2. No
3. 3. I am unsure

Which of the following therapeutic services does your child receive? (please select all that apply)

1. 1. Physiotherapy
2. 2. Occupational therapy
3. 3. Speech therapy
4. 4. Behavior Modification
5. 5. Psychotherapy
6. 6. Other
7. 7. None

Are you a mother or a father?

1. 1. Mother
2. 2. Father

How much time, on average, do you devote to your child of determination every day?

1. 1. No time
2. 2. Less than 30 minutes
3. 3. 30-45 minutes
4. 4. 45 minutes – 1 hour
5. 5. 1 – 1.5 hours
6. 6. 1.5 – 2 hours
7. 7. More than 2 hours

How many days of the week, on average, do you spend time with your child of determination?

1. 1. 0
2. 2. 1
3. 3. 2
4. 4. 3
5. 5. 4
6. 6. 5
7. 7. 6
8. 8. 7

In this section, please rate each of these statements as it relates to your involvement and perceptions in raising of your child of determination.

|  | Strongly Agree | Agree | Neutral | Disagree | Strongly Disagree |
| --- | --- | --- | --- | --- | --- |
| I share the responsibility of raising my child of determination. | ❏ | ❏ | ❏ | ❏ | ❏ |
| I am aware of my child of determination’s mental and cognitive skills | ❏ | ❏ | ❏ | ❏ | ❏ |
| I am supportive to my child of determination’s mental and cognitive skills | ❏ | ❏ | ❏ | ❏ | ❏ |
| I respect the individuality and privacy of my child of determination | ❏ | ❏ | ❏ | ❏ | ❏ |
| I am aware of how to take care of my child of determination | ❏ | ❏ | ❏ | ❏ | ❏ |
| I dress my child of determination or help him/her to get dressed. | ❏ | ❏ | ❏ | ❏ | ❏ |
| I eat meals with my child of determination | ❏ | ❏ | ❏ | ❏ | ❏ |
| I participate in providing financial support for my child of determination, such as money and housing. | ❏ | ❏ | ❏ | ❏ | ❏ |
| I participate in providing basic needs for my child of determination, such as food, drinks, and clothing, etc. | ❏ | ❏ | ❏ | ❏ | ❏ |
| I have a good relationship with my child of determination. | ❏ | ❏ | ❏ | ❏ | ❏ |
| I interact with my child of determination with warmth and love. | ❏ | ❏ | ❏ | ❏ | ❏ |
| I hug and/or kiss my child of determination | ❏ | ❏ | ❏ | ❏ | ❏ |
| I laugh with my child of determination | ❏ | ❏ | ❏ | ❏ | ❏ |
| I comfort my child of determination when they are upset or crying | ❏ | ❏ | ❏ | ❏ | ❏ |
| I tell my child of determination that I love them | ❏ | ❏ | ❏ | ❏ | ❏ |
| My child of determination feels comfortable and at ease around me | ❏ | ❏ | ❏ | ❏ | ❏ |
| I talk to and chat with our child of determination | ❏ | ❏ | ❏ | ❏ | ❏ |
| I am accepting of any kind of conversation with my child of determination, no matter what the subject is. | ❏ | ❏ | ❏ | ❏ | ❏ |
| I support in teaching and guiding my child of determination on what is right and wrong. | ❏ | ❏ | ❏ | ❏ | ❏ |
| I help my child of determination learn new things and develop their skills. | ❏ | ❏ | ❏ | ❏ | ❏ |
| I assist my child of determination with homework, lessons, and tasks. | ❏ | ❏ | ❏ | ❏ | ❏ |
| I support my child of determination and assist them in difficult situations. | ❏ | ❏ | ❏ | ❏ | ❏ |
| I teach religious principles to my child of determination (For example teaching prayer and memorizing the surahs of the Holy Quran). | ❏ | ❏ | ❏ | ❏ | ❏ |
| My child of determination trusts that I am a source of healthy and positive support when dealing with challenges and crises. | ❏ | ❏ | ❏ | ❏ | ❏ |
| I am concerned about the mental health of my child of determination. | ❏ | ❏ | ❏ | ❏ | ❏ |
| I am concerned about the physical health of my child of determination. | ❏ | ❏ | ❏ | ❏ | ❏ |
| I am aware of my child of determination’s ambitions. | ❏ | ❏ | ❏ | ❏ | ❏ |
| I make an effort to support the development of my child of determination. | ❏ | ❏ | ❏ | ❏ | ❏ |
| I recognize the skills of my child of determination | ❏ | ❏ | ❏ | ❏ | ❏ |
| I encourage and praise my child of determination | ❏ | ❏ | ❏ | ❏ | ❏ |
| I accept the actions and behaviors of my child of determination. | ❏ | ❏ | ❏ | ❏ | ❏ |
| I am aware of the interests of my child of determination. | ❏ | ❏ | ❏ | ❏ | ❏ |
| I take my child of determination to the shops. | ❏ | ❏ | ❏ | ❏ | ❏ |
| I take my child of determination to centers/schools/clinics. | ❏ | ❏ | ❏ | ❏ | ❏ |
| I take my child of determination to fun activities such as the zoo or a sporting event. | ❏ | ❏ | ❏ | ❏ | ❏ |
| I play with toys or puzzles with my child of determination. | ❏ | ❏ | ❏ | ❏ | ❏ |
| I interact physically with my child of determination, such as by roughhousing or tickling. | ❏ | ❏ | ❏ | ❏ | ❏ |

In this section, please rate each of these statements as it relates to your involvement in supporting the raising of your child of determination.

|  | Strongly disagree | Disagree | Neutral | Agree | Strongly agree |
| --- | --- | --- | --- | --- | --- |
| One of the most challenging aspects of my life is being a father of a child of determination. | ❏ | ❏ | ❏ | ❏ | ❏ |
| I try my best to raise my child of determination, and am keen on continuously learning more skills about the different stages of my child's development. | ❏ | ❏ | ❏ | ❏ | ❏ |
| I realize that my child of determination needs more support than typical children. | ❏ | ❏ | ❏ | ❏ | ❏ |
| I participate in collaboration and dialogue with my child of determination's mother about raising our child. | ❏ | ❏ | ❏ | ❏ | ❏ |
| I put so much into parenting my child of determination and don't have time for myself. | ❏ | ❏ | ❏ | ❏ | ❏ |
| I feel overwhelmed with my responsibilities toward my child of determination. | ❏ | ❏ | ❏ | ❏ | ❏ |
| I believe that my child's mother is more friendly and patient towards our child of determination than I am, and she needs continuous support and motivation to cope with raising our child of determination. | ❏ | ❏ | ❏ | ❏ | ❏ |
| I coordinate with my child of determination's mother regarding how our child should be raised. | ❏ | ❏ | ❏ | ❏ | ❏ |
| I share with my child of determination's what is going on in his mind at any time about our child. | ❏ | ❏ | ❏ | ❏ | ❏ |
| My commitment to providing proper care for my child of determination makes me a good father. | ❏ | ❏ | ❏ | ❏ | ❏ |
| My top priority is raising my child of determination, as I am an authority figure in the family. | ❏ | ❏ | ❏ | ❏ | ❏ |
| My involvement in parenting my child of determination doesn't interfere or conflict with their mother's parenting style. | ❏ | ❏ | ❏ | ❏ | ❏ |
| My involvement in raising our child of determination helps facilitate their mother's roles and responsibilities. | ❏ | ❏ | ❏ | ❏ | ❏ |
| I disagree with my child of determination's mother about how long I should interact with our child. | ❏ | ❏ | ❏ | ❏ | ❏ |
| I am satisfied with my involvement as a father of a child of determination. | ❏ | ❏ | ❏ | ❏ | ❏ |

Have you ever attended or participated in evidence-based training programs (for example, Applied Behavior Analysis, Picture Exchange Communication System, or TEACCH Program)?

1. 1. Yes
2. 2. No
3. 3. I am unsure

In this section, please rate each of these statements as it relates to your experience with evidence-based programs

|  | Strongly disagree | Disagree | Neutral | Agree | Strongly agree |
| --- | --- | --- | --- | --- | --- |
| Attending evidence-based programs (for example, Applied Behavior Analysis, Picture Exchange Communication System, or TEACCH Program) provided me with helpful information towards raising my child of determination. | ❏ | ❏ | ❏ | ❏ | ❏ |
| Attending evidence-based programs helped me develop and learn communication skills suitable for my child of determination. | ❏ | ❏ | ❏ | ❏ | ❏ |
| Attending evidence-based programs helped me use appropriate strategies to support the development of my child of determination. | ❏ | ❏ | ❏ | ❏ | ❏ |
| Attending evidence-based programs enabled me to overcome challenges of raising my child of determination. | ❏ | ❏ | ❏ | ❏ | ❏ |
| Attending evidence-based programs has increased my ability to meet the needs of my child of determination and enhance their capabilities. | ❏ | ❏ | ❏ | ❏ | ❏ |
| Attending evidence-based programs contributed to increasing my participation with my child of determination's mother in raising our child. | ❏ | ❏ | ❏ | ❏ | ❏ |
| Attending an evidence-based training program helped me add new knowledge that enhanced my ability to raise my child of determination. | ❏ | ❏ | ❏ | ❏ | ❏ |
